# The chaperone GRP94 interacts with the proprotein convertase furin and regulates TGF-beta maturation in human primary M2 macrophages

**DOI:** 10.1038/s41420-025-02866-2

**Published:** 2025-12-15

**Authors:** Valentin Baverel, Fangmin Wang, Carmen Garrido, Evelyne Kohli

**Affiliations:** 1 Université Bourgogne Europe, UFR des Sciences de Santé, Dijon, France; 2 UMR INSERM/uB/AGROSUP 1231, Team HSP-Pathies, labellisée Ligue Nationale contre le Cancer and Laboratoire d’Excellence LipSTIC, Dijon, France; 3 Zhejiang Provincial Key Lab of Addiction, Ningbo Kangning Hospital, School of Medicine, Ningbo University, Ningbo, China; 4 Centre anti-cancéreux Georges François Leclerc, Dijon, France; 5 CHU, Dijon, France

**Keywords:** Tumour immunology, Breast cancer, Innate immunity, Transforming growth factor beta, Monocytes and macrophages

## Abstract

Transforming growth factor-beta (TGFβ) is a major immunosuppressive cytokine produced by various cell types, including regulatory T cells (Tregs) and M2 macrophages. In Tregs, GRP94 is known as a key protein regulating TGFβ expression by chaperoning both the TGFβ docking receptor, GARP, and the integrin αvβ8. We previously reported that GRP94 inhibition in a triple-negative breast cancer (TNBC) murine model induced a decrease in intra-tumoral CD206 + M2-like macrophages that correlated with a decrease in collagen content, an increase in CD8+ cells in tumors and a reduced tumor volume. Here, we investigated the impact of GRP94 inhibition on TGFβ expression focusing on a possible interaction with furin, a proprotein convertase responsible for the first step in TGFβ maturation. We demonstrated in human primary M2 macrophages that GRP94 interacted with furin and that GRP94 inhibition by its selective inhibitor PU-WS13 led to a decrease in furin enzymatic activity, which was associated with a decrease in TGFβ secretion. Similar results were obtained in the human TNBC cell line MDA-MB-231 using PU-WS13 and GRP94 inhibitor- 1, suggesting that our findings are not cell type-specific. Finally, we showed that GRP94 associated with LRRC33, a GARP paralog in macrophages. Together, these findings support the hypothesis that GRP94 plays a key role in regulating the TGFβ maturation pathway, not only in Tregs as previously reported, but also in M2 macrophages and tumor cells.

## Introduction

Glucose-Regulated Protein 94 (GRP94), also known as Glycoprotein 96 (Gp96), is an endoplasmic reticulum chaperone, member of the heat shock protein 90 family. It chaperones two proteins involved in the activation of transforming growth factor-beta (TGFβ) in regulatory T cells (Tregs): the TGFβ docking receptor Glycoprotein A Repetitions Predominant (GARP) and integrin αvβ8, thus controlling Treg maintenance and function [1–3]. Previous studies have investigated the role of GRP94 in macrophages and shown that it may also be a key player in immunosuppressive M2 macrophages, both in vitro and in vivo. Schreiter et al (2005) first reported that GRP94 was associated with gut microbiota immune tolerance, with its expression being strongly induced during monocyte differentiation into intestinal macrophages in healthy patients but not in patients with Crohn’s disease [4]. We have recently shown that GRP94 was expressed at the membrane of human primary M2 but not M1 macrophages and was overexpressed by a subset of CD206 + M2-like macrophages infiltrating murine triple-negative breast 4T1 tumors [5]. Moreover, its inhibition by a selective chemical inhibitor, PU-WS13, in this model, induced a decrease in intra-tumoral CD206 + M2-like macrophages, which correlated with decreased collagen content, an increase in CD8+ cells in the tumor microenvironment, and a reduction in tumor volume [6].

TGFβ is a major immunosuppressive cytokine controlling immune cell responses and playing critical roles in numerous biological processes [7], making it a key player in diseases such as fibrosis and cancer [8]. Among cells producing this cytokine, the anti-inflammatory M2-like macrophages are well known for their important role in the tumor microenvironment [9]. TGFβ favors the formation of macrophages with an anti-inflammatory M2-like phenotype which in turn produce TGFβ, promoting tumor progression through stimulation of angiogenesis, metastasis and suppression of anti-tumoral immunity [7, 10]. Considering that TGFβ is a major immunosuppressive cytokine produced by M2-like macrophages [11], and that GRP94 has a critical role in the production of active TGFβ in Tregs [2], we hypothesized that our results showing that GRP94 inhibition decreased intra-tumoral M2-like macrophages in a 4T1 TNBC model could be linked to an impact of GRP94 inhibition on the production of active TGFβ.

TGFβ is produced as an inactive precursor, pro-TGFβ, that needs to be cleaved in the Golgi apparatus by furin, a proprotein convertase involved in the cleavage of precursor proteins containing the recognition motif R/K-X_n_-R/K↓ [12–14]. After this first cleavage step, active TGFβ fragment remains non-covalently bound to the latency-associated peptide to form the latent TGFβ (LTGFβ), which can either associate a) with latent TGFβ binding proteins (LTBPs) to be secreted to the extracellular matrix or b) with an anchorage protein for cell surface expression, GARP, a client of GRP94 in platelets and regulatory T cells, or c) with the leucine-rich repeat-containing protein 33 (LRRC33), a GARP paralog in macrophages [10, 15, 16]. Active TGFβ is finally released from its latent form by integrin αvβ8, also a client of GRP94, and metalloproteinases, like the matrix metalloproteinase 14 (MMP14) [1, 8, 17].

In this work, to study the effect of GRP94 on mature TGFβ production, we used two chemicals reported to selectively inhibit GRP94 and that, in contrast to gene depletion approaches, are not known to induce a compensatory expression of other chaperones, especially from the Hsp70 family [18–20]. We found that inhibition of GRP94 decreased the production of mature TGFβ in human primary M2 macrophages differentiated from PBMC of healthy donors. As we previously found that GARP was not expressed at the membrane of human primary M2 macrophages [5], we hypothesized that GRP94 impacted other proteins involved in the maturation of TGFβ in macrophages, knowing that integrin αvβ8 is a client of GRP94 [1]. We demonstrated that GRP94 interacted with furin, the enzyme involved in the early cleavage of pro-TGFβ, and inhibited its enzymatic activity. We confirmed these results in the human triple negative breast cancer (TNBC) cell line MDA-MB-231. Finally, we showed that GRP94 also associated with LRRC33 in M2 macrophages, indicating that this chaperone could control the entire TGFβ maturation pathway.

## Results

### The GRP94 inhibitor PU-WS13 decreases the production of mature TGFβ in human primary M2 macrophages

**Fig. 1:**
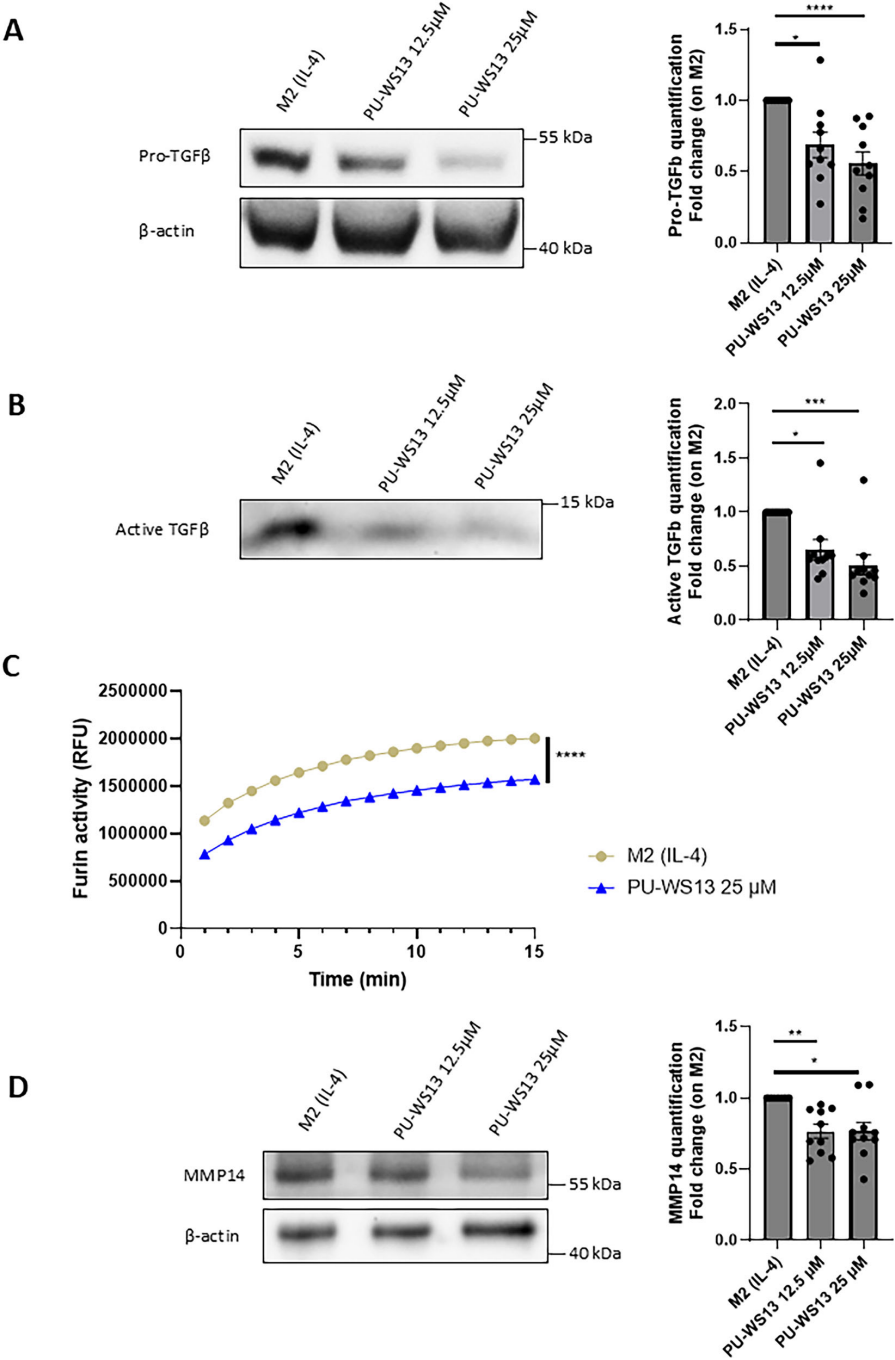
GRP94 inhibition impacts furin enzymatic activity and the expression of furin substrates TGFβ and MMP14 in M2 macrophages. PBMC of healthy volunteers differentiated in M2 macrophages and activated with IL-4 were treated with PU-WS13 (12.5 or 25 µM) during all the activation period. Western-blot analysis of pro-TGFβ expression in cell lysates (**A**) and active TGFβ secretion in supernatants (**B**) from M2 macrophages treated or not with PU-WS13 during 24 h (representative images, *n* = 10 for each experiment) (**C**) Furin enzymatic activity analysis in cell lysates of M2 macrophages treated or not 24 h with PU-WS13 25 µM (*n* = 5). Fluorescence signals (expressed in relative fluorescence units, RFU) due to cleavage of the furin fluorogenic substrate, pERTKR-AMC, in cell lysates were recorded during 15 min (**D**) Western- blot analysis of MMP14 expression in M2 macrophages cell lysates (representative image, *n* = 10). (*p < 0.05; **p < 0.01; ***p < 0.001; ****p < 0.0001).

To investigate the impact of GRP94 inhibition by PU-WS13 on TGFβ production by human primary M2 macrophages, we analyzed TGFβ expression in cell lysates and supernatants from macrophages differentiated from PBMC of healthy donors. M2 macrophages were activated by M-CSF and IL-4 and treated or not with PU-WS13 during 24 h. The M2 macrophage phenotype was validated through STAT6 phosphorylation (Fig. S1). As shown in Fig. 1, GRP94 inhibition by PU-WS13 at the doses of 12.5 and 25 µM induced a significant decrease in pro-TGFβ expression in cell lysates (Fig. 1A) (*P* = 0.0253 and 0.0001 for PU-WS13 12.5 and 25 µM respectively) and in active TGFβ secretion in M2 macrophages supernatants (Fig. 1B) (*P* = 0.0442 and 0.0008 for PU-WS13 12.5 and 25 µM respectively). This effect of PU-WS13 on TGFβ expression could not be explained by a cytotoxic effect at the tested doses ([5] and Fig. S2). Moreover, PU-WS13 at 25 µM did not significantly impact TGFβ mRNA (Fig. S3), meaning that GRP94 inhibition by PU-WS13 in M2 macrophages likely affects TGFβ maturation rather than its transcription.

### GRP94 inhibition decreases the activity of the proprotein convertase furin and the expression of the metalloproteinase MMP14

Considering that furin is involved in the early cleavage of pro-TGFβ into LTGFβ [7, 12], we hypothesized that our results could be explained by an effect of GRP94 inhibition in pro-TGFβ cleavage. Although PU-WS13 at the dose of 25 µM did not seem to significantly affect furin expression (Fig S4), it did induce a decrease in furin enzymatic activity (Fig. 1C, *P* < 0.0001 at 15 min). To further investigate the impact of the decreased furin enzymatic activity in M2 macrophages treated with PU-WS13, we analyzed the expression of MMP14, a metalloproteinase implicated in the final activation step of TGFβ that also requires cleavage by furin during its processing [17, 21]. We found a significant decrease in MMP14 expression in cell lysates from M2 macrophages treated with PU-WS13 at the tested doses (Fig. 1D, *P* = 0.0037 and *P* = 0.0139 for PU-WS13 12.5 µM and 25 µM respectively). This decrease could not be explained by an impact on MMP14 mRNA (Fig. S3).

These results indicate that GRP94 inhibition leads to a decrease in furin enzymatic activity, which is consistent with a reduction in the expression of the active forms of TGFβ and MMP14, both requiring furin for their maturation.

### GRP94 interacts with furin in human primary M2 macrophages

**Fig. 2:**
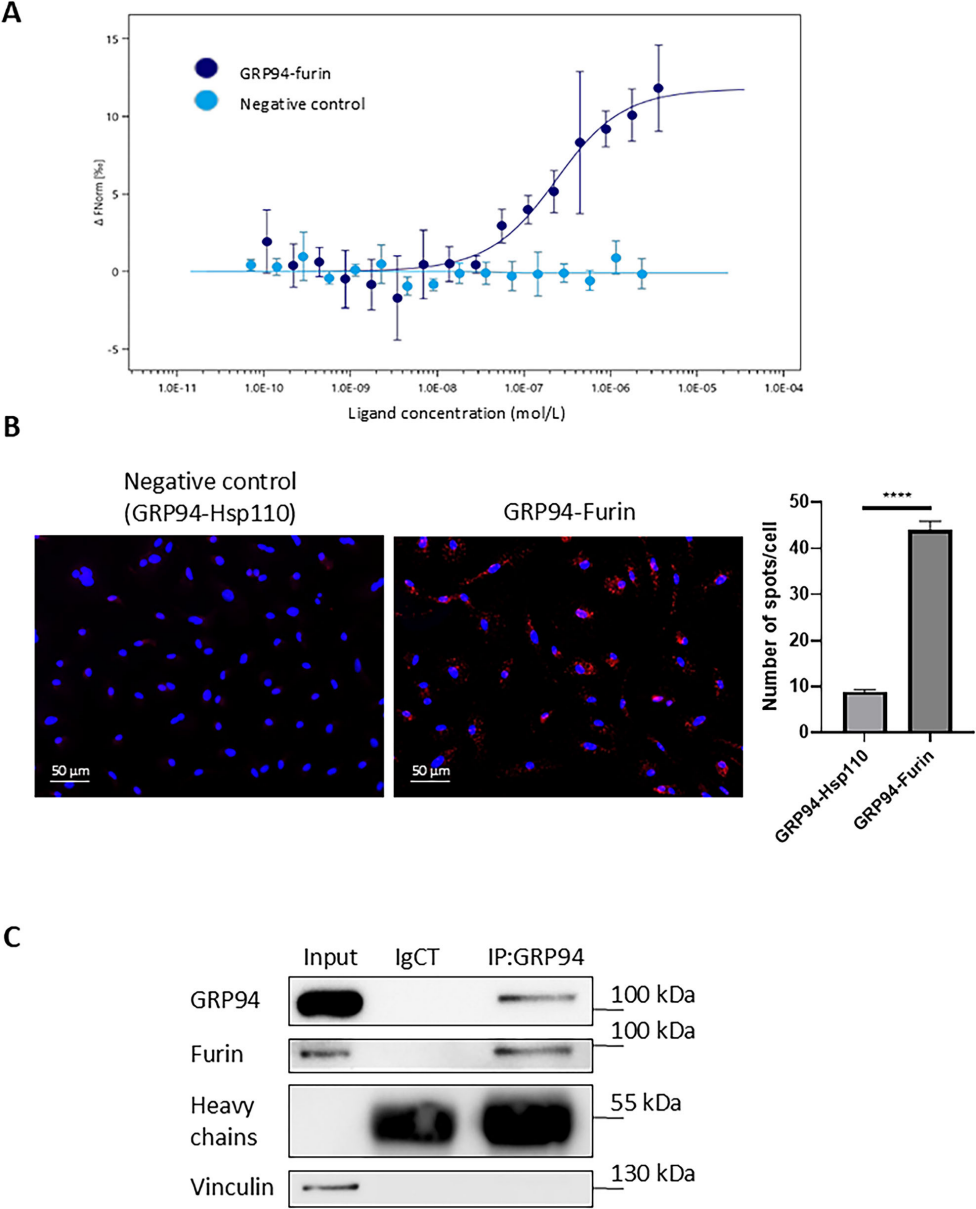
GRP94 interacts with furin in M2 macrophages. **A** Analysis by microscale thermophoresis of the specific binding between Nano-RED-labelled recombinant GRP94 (100 nM) and either recombinant furin (3.58 μM to 0.1 nM) or the negative control recombinant Hsp110 (2.33 μM to 0.07 nM). Changes in thermophoresis depending on ligand (furin or Hsp110) concentration from *n* = 3 experiments were plotted and expressed as ∆Fnorm (‰). **B** Staining and quantification of proximity ligation assays (Duolink™ PLA) of GRP94 and furin or the negative control Hsp110 in human PBMC-derived M2 macrophages (*n* = 3 donors). Scale bars = 50 µm. The bar plot represents the mean number of spots for each cell analyzed +/− SEM. (****p < 0.0001). **C** GRP94-furin co-immunoprecipitation in M2 macrophages. GRP94 was immunoprecipitated in M2 macrophages cell lysates using an anti-GRP94 antibody (IP:GRP94) or a non-relevant antibody (IgCT). Samples were analyzed by western-blot (representative images, *n* = 7).

To better understand how PU-WS13 inhibits furin enzymatic activity, we hypothesized a possible interaction between GRP94 and furin. We first investigated the binding between recombinant GRP94 and furin using microscale thermophoresis (Nanotemper™ technology). As shown in Fig. 2A, we found an interaction with a Kd = 186 nM (+/− 63 nM). Proximity ligation assay (Duolink PLA™) showed that this association took also place *in cellulo*, as indicated by the increased number of spots compared to the negative control (Fig. 2B, *P* < 0.0001). Finally, co-immunoprecipitation experiments confirmed GRP94 and furin interaction (Fig. 2C).

These results show that GRP94 interacts with furin in human primary M2 macrophages, consistent with the fact that its inhibition decreases furin activity as well as the production of mature TGFβ and MMP14.

### GRP94 also interacts with furin in the human TNBC cell line MDA-MB-231 and its inhibition impacts TGFβ maturation

**Fig. 3:**
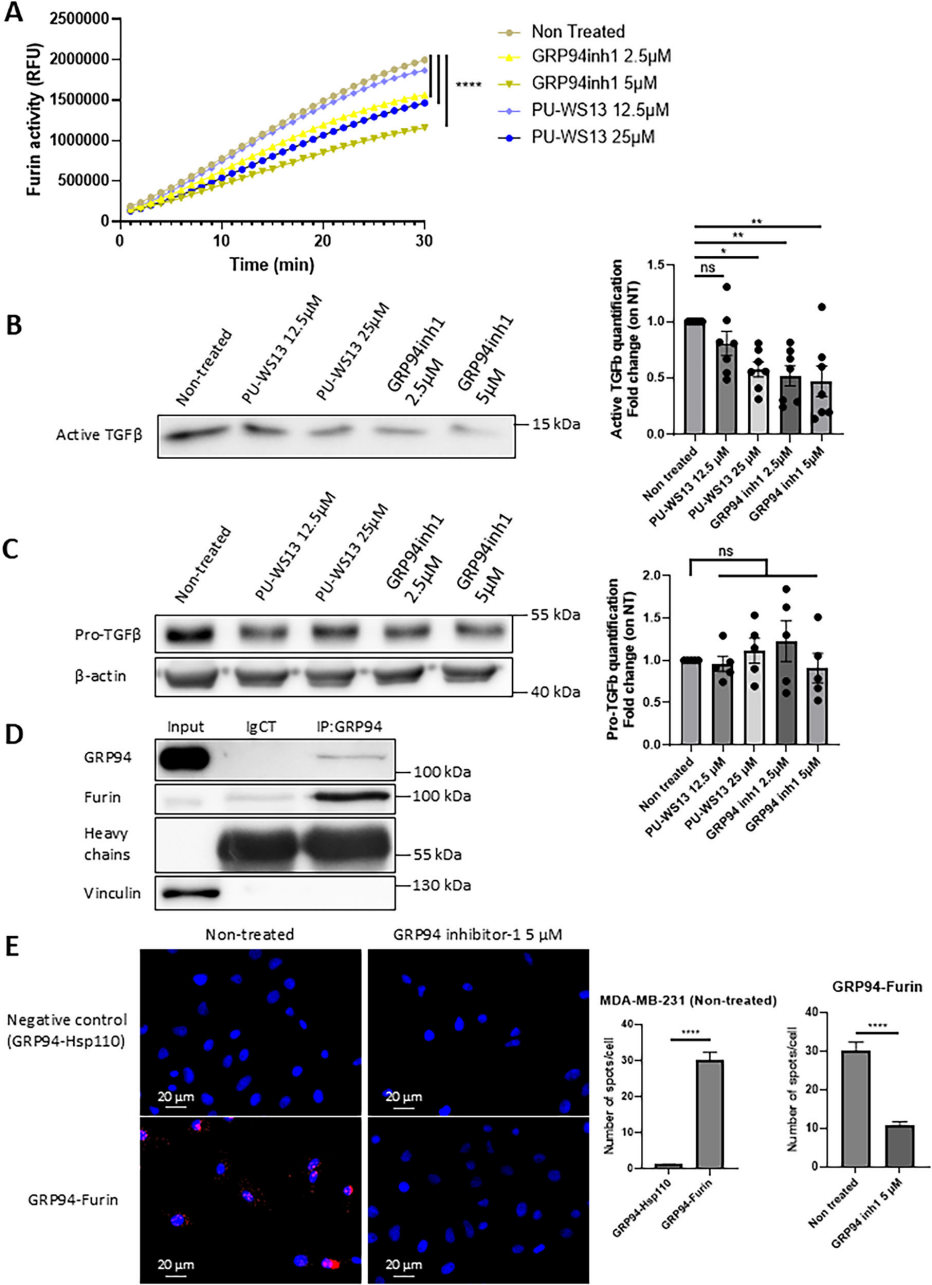
GRP94 interacts with furin and GRP94 inhibition impacts TGFβ expression in MDA-MB-231 cells. **A** Furin enzymatic activity analysis in cell lysates of MDA-MB-231 treated or not 24 h with GRP94 inhibitors, PU-WS13 12.5 or 25 µM or GRP94 inhibitor-1 (GRP94inh1) 2.5 or 5 µM (*n* = 6). Fluorescence signal (expressed in relative fluorescence units, RFU) due to cleavage of the furin fluorogenic substrate, pERTKR-AMC, in cell lysates was recorded during 30 min. (****p < 0.0001). Western- blot analysis of (**B**) active TGFβ secretion in supernatants (representative images, *n* = 7) and (**C**) pro-TGFβ expression in cell lysates (representative images, *n* = 5) from MDA-MB-231 treated or not 24 h with GRP94 inhibitors, PU-WS13 12.5 or 25 µM or GRP94 inhibitor-1 (GRP94inh1) 2.5 or 5 µM. (*p < 0.05; **p < 0.01). **D** GRP94-furin co-immunoprecipitation in MDA-MB-231 cells. GRP94 was immunoprecipitated in MDA-MB-231 cell lysates using an anti-GRP94 antibody (IP:GRP94) or a non-relevant antibody (IgCT). Samples were analyzed by western-blot (representative images, *n* = 4). **E** Staining and quantification of proximity ligation assays (Duolink™ PLA) of GRP94 and furin or the negative control Hsp110 in MDA-MB-231 cells treated or not 24 h with GRP94 inhibitor-1 5 µM (*n* = 3 experiments). Scale bars = 20 µm. The bar plot represents the mean number of spots for each cell analyzed +/− SEM. (****p < 0.0001).

**Fig. 4:**
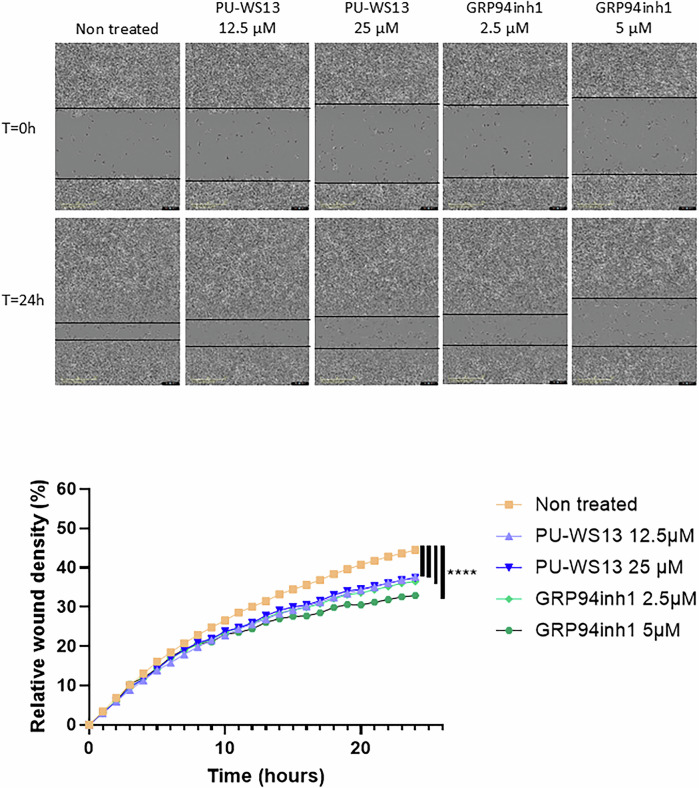
GRP94 inhibition impacts MDA-MB-231 cell migration. Impact of GRP94 inhibitors, PU-WS13 12.5 or 25 µM and GRP94 inhibitor-1 (GRP94inh1) 2.5 or 5 µM, on MDA-MB-231 cell migration was analyzed by wound healing assay. Wounds were realized in starved cells at confluency and treatments added in serum free medium. Using an Incucyte® S3 Live-Cell Analysis System, images of the wound areas were taken every hour during 24 h. Representative images at T = 0 h and T = 24 h of *n* = 6 experiments are shown in the upper panel with black bars highlighting the wound borders. Scale bars = 400 µm. Data are represented as relative wound densities over time in the lower panel (*n* = 6) (****p < 0.0001).

TGFβ and furin are key proteins involved in tumor progression and can be produced by intra-tumoral immune cells such as M2 macrophages as well as by tumor cells themselves [8, 22]. To complete our understanding of the effect of GRP94 inhibition in the reduction of tumor volumes previously observed in our TNBC model [6], we used the human TNBC cell line MDA-MB-231 to investigate whether the results obtained in M2 macrophages could be reproduced in these tumor cells. For these experiments, we used an additional selective GRP94 inhibitor, the GRP94 inhibitor-1, a benzamide compound [18]. As for M2 macrophages, GRP94 inhibition by both PU-WS13 at 25 µM and GRP94 inhibitor-1 at 2.5 and 5 µM decreased furin enzymatic activity in cell lysates (Fig. 3A) (*P* < 0.0001 at 30 min for PU-WS13 25 µM and GRP94 inhibitor-1 2.5 and 5 µM) as well as the production of active TGFβ in the cells’ supernatants (Fig. 3B) (*P* = 0.0112, 0.0041 and 0.0023 for PU-WS13 25 µM, GRP94 inhibitor-1 2.5 and 5 µM respectively). However, contrary to M2 macrophages, neither PU-WS13 nor GRP94 inhibitor-1 decreased significantly pro-TGFβ expression in MDA-MB-231 cell lysates (Fig. 3C). Of note, both inhibitors did not exhibit cytotoxicity on MDA-MB-231 cells at the tested doses (Fig. S5). As in M2 macrophages, we also co-immunoprecipitated GRP94 and furin in MDA-MB-231 cells (Fig. 3D). Proximity ligation assay (Duolink PLA™) also showed that GRP94 and furin are associated in MDA-MB-231 cells (Fig. 3E, *P* < 0.0001), and GRP94 inhibitior-1 at the dose of 5 µM decreased this association (Fig. 3E, *P* < 0.0001). Finally, we used a wound healing assay to confirm that the decrease in mature TGFβ correlated with a decrease in its function, and showed that GRP94 inhibition by PU-WS13 or GRP94 inhibitor-1 significantly decreased MDA-MB-231 migration for all tested doses (Fig. 4, *P* < 0.0001 for all tested conditions). Together, our results in MDA-MB-231 confirm those obtained in M2 macrophages showing that GRP94-furin interaction is associated with a decrease in the production of mature TGFβ, which correlated with a functional impact, i.e., cell migration.

### GRP94 also associates with LRRC33, another protein involved in the TGFβ maturation pathway

**Fig. 5:**
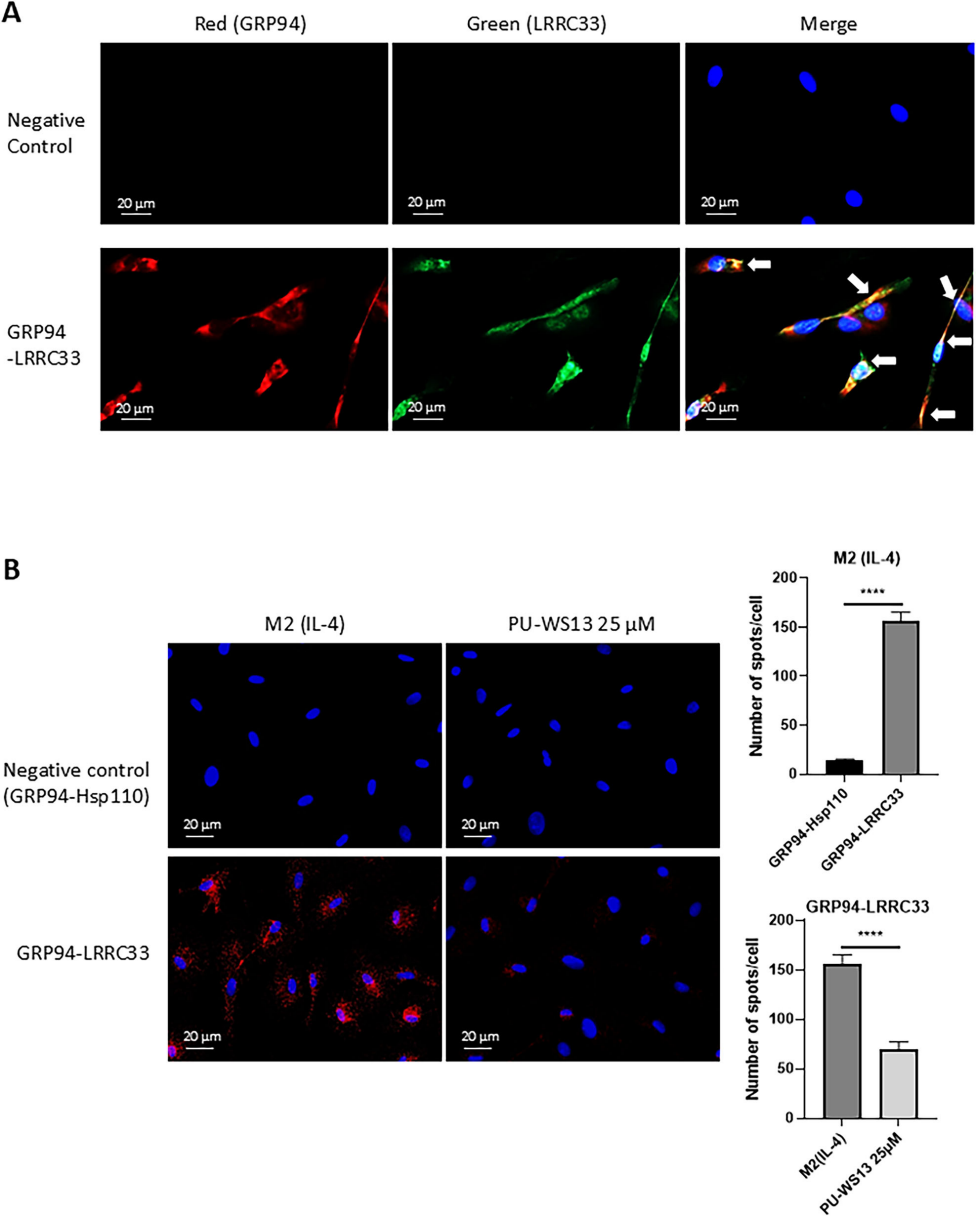
GRP94 associates with LRRC33, another protein involved in the TGFβ maturation pathway in M2 macrophages. **A** Immunofluorescence staining of GRP94 (red channel) and LRRC33 (green channel) in human PBMC-derived M2 macrophages. Cells stained without primary antibodies were used as negative control. White arrows indicate colocalization areas. Scale bars = 20 µm. Representative images of *n* = 3 experiments. **B** Staining and quantification of proximity ligation assays (Duolink™ PLA) of GRP94 and LRRC33 or the negative control Hsp110 in human PBMC-derived M2 macrophages treated or not with PU-WS13 25 µM (*n* = 6 donors). Scale bars = 20 µm. The bar plot represents the mean number of spots for each cell analyzed +/− SEM. (****p < 0.0001).

Considering that GRP94 interacts with GARP in Tregs [2], we decided to investigate a possible association between GRP94 and LRRC33, a GARP paralog in macrophages [15]. As shown in Fig. 5, we found by immunofluorescence staining colocalization areas between GRP94 and LRRC33 (Fig. 5A) in M2 macrophages. Using proximity ligation assay (Duolink PLA™), we also showed a proximity between GRP94 and LRRC33 (Fig. 5B) (*P* < 0.0001 compared to the negative control). Moreover, using the same method, we showed a decrease in the proximity between GRP94 and LRRC33 in M2 macrophages treated with PU-WS13 25 µM (Fig. 5B) (*P* < 0.0001).

Altogether, our results indicate that GRP94 may associate with LRRC33 in M2 macrophages, suggesting that GRP94 could be implicated at several steps of TGFβ maturation pathway.

## Discussion

In a previous study, we demonstrated in a 4T1 TNBC murine model that GRP94 inhibition by the selective inhibitor PU-WS13 induced a reduction in intra-tumoral CD206 + M2-like macrophages. This was associated with decreased collagen content, an increase in CD8+ cells in the tumor microenvironment, and in tumor regression [6]. TGFβ is a major immunosuppressive cytokine produced by various cell types in the tumor microenvironment, including tumor-associated M2-like macrophages [8]. On the one hand, TGFβ is known for promoting lymphocyte differentiation into Tregs and suppressing CD8+ cytotoxic lymphocyte responses and also to mediate collagen production [23]. On the other hand, GRP94 is a chaperone for both GARP [2] and integrin αvβ8 [1], two proteins involved in the TGFβ maturation pathway in Tregs [3]. Consequently, we hypothesized that our previous findings showing PU-WS13 antitumor effects in the TNBC 4T1 model could result from GRP94 inhibition effect on TGFβ production in M2-like macrophages. In this study, we confirmed this hypothesis by showing that GRP94 inhibition in human PBMC-derived M2 macrophages by PU-WS13 led to a dose-dependent decrease in the expression of active TGFβ.

To further investigate how GRP94 inhibition could impact TGFβ secretion, we studied the interaction of GRP94 with furin, a proprotein convertase responsible for the first step of TGFβ maturation through the cleavage of pro-TGFβ into LTGFβ [7, 12]. We first showed extracellularly that GRP94 directly interacts with furin using microscale thermophoresis. This GRP94-furin interaction in vitro was further confirmed in human primary M2 macrophages by proximity ligation assay (PLA) and co-immunoprecipitation. Moreover, GRP94 inhibition by PU-WS13 reduced furin enzymatic activity but not its expression in M2 macrophages, indicating that the GRP94-furin interaction is not necessary for correct protein folding in the ER but is essential for its enzymatic activity. Surprisingly, while GRP94 inhibition decreased furin enzymatic activity and active TGFβ secretion, we found that pro-TGFβ levels were also decreased in the macrophage lysates when a decrease in its processing by furin should have resulted in an increase [12]. Since TGFβ transcription was not affected by PU-WS13 treatment, a possible explanation for this decrease in non-cleaved pro-TGFβ might be its degradation.

To confirm that the impact of GRP94 inhibition on furin enzymatic activity is not restricted to TGFβ, we analyzed the expression of MMP14, a metalloproteinase also requiring furin for its processing [21]. As with active TGFβ in supernatants, GRP94 inhibition by PU-WS13 significatively decreased MMP14 expression in human PBMC-derived M2 macrophages. Since both TGFβ and MMP14 require furin for their processing [12, 21], we can infer that the decreased expression of both proteins is due to the reduced furin enzymatic activity resulting from GRP94 inhibition by PU-WS13 in human primary M2 macrophages. However, since MMP14 is also involved in TGFβ activation [17], it appears that the effect of GRP94 inhibition on active TGFβ may not be limited to a direct impact on its processing by furin, but may also involve indirect effects through the decreased expression of MMP14.

To better understand the results obtained in our TNBC murine model [6] and considering that cancer cells can also produce TGFβ [8], we investigated the impact of GRP94 inhibition on TGFβ activation in the human TNBC cell line MDA-MB-231. Using co-immunoprecipitation and PLA, we showed that GRP94 also interacts with furin in MDA-MB-231 cells. Moreover, GRP94 inhibitor-1, another selective inhibitor with a benzamide scaffold distinct from the purine scaffold of PU-WS13 [18, 20], decreased the association between GRP94 and furin, indicating that the inhibitor could impede the interaction. As observed with PU-WS13 in M2 macrophages, both PU-WS13 and GRP94 inhibitor-1 decreased furin enzymatic activity and active TGFβ secretion. In line with this result, a wound healing assay showed decreased cell migration in MDA-MB-231 treated with GRP94 inhibitors, which correlated with decreased TGFβ secretion in the supernatants, TGFβ being an inducer of epithelial-to-mesenchymal transition [8]. In contrast to M2 macrophages, pro-TGFβ levels were not decreased in the cell lysates of MDA-MB-231 treated with GRP94 inhibitors suggesting that the degradation rate of non-cleaved TGFβ could vary depending on the cell type.

Based on those results and considering that Koo et al (2010) have previously shown that GRP94 could form an immunoprecipitable complex with furin in HEK293F cells [24], we can hypothesize that this interaction is not cell-type dependent. These findings bring a new insight on the major role of GRP94 in cancer but also in a variety of diseases involving proteins processed by furin, including growth factors such as insulin growth factors and their receptors, metalloproteinases like MMP2 or viral glycoproteins such as the SARS-CoV-2 Spike glycoprotein [14, 22, 25].

In this work we focused our investigations on the role of GRP94 in TGFβ maturation through its interaction with furin. Nevertheless, GRP94 is also known as a chaperone of two other proteins involved in the TGFβ maturation process in Tregs, integrin αvβ8 and GARP [1–3]. GARP is not expressed on the membrane of M2 macrophages, either murine [26] or human [5], but several studies have shown that macrophages express LRRC33, a paralog of GARP, which also acts as a docking receptor for integrin-dependent TGFβ activation [15, 16, 27]. To further investigate the role of GRP94 in TGFβ maturation in human primary M2 macrophages, we investigated the association between GRP94 and LRRC33. We observed colocalization and proximity between these proteins. Although our results do not conclusively show that GRP94 acts as a chaperone of LRRC33, they indicate that GRP94 and LRRC33 may be associated in macrophages. Furthermore, we demonstrated that their proximity was decreased by GRP94 inhibition by PU-WS13 in M2 macrophages. Therefore, GRP94 inhibition by PU-WS13 may impact active TGFβ secretion by M2 macrophages not only through its impact on furin activity but also through LRRC33. Since GRP94 is already known as a chaperone of integrin αvβ8 [1], our results suggest that, through its interactions with furin and possibly with LRRC33, GRP94 could be a key regulatory protein controlling the entire TGFβ maturation pathway in M2 macrophages. This is consistent with our previous in vivo findings in the 4T1 model, which demonstrate an increase in CD8+ cells accompanied by a decrease in collagen content following treatment of mice with PU-WS13 [6]. Additional in vitro studies are needed to clarify the effects of GRP94 inhibition on the crosstalk between macrophages and other cells in the tumor microenvironment, such as cytotoxic T cells, regulatory T cells, and cancer-associated fibroblasts.

In conclusion, this study demonstrates that GRP94 interacts with the proprotein convertase furin in human primary M2 macrophages. Inhibition of GRP94 with the selective inhibitor PU-WS13 resulted in a reduction of furin enzymatic activity, as well as a decrease in the expression of active TGFβ in supernatants and MMP14 in cell lysates, both proteins requiring furin for their maturation. Similar findings were observed in the human TNBC cell line MDA-MB-231, using both PU-WS13 and another selective inhibitor, GRP94 inhibitor-1. Additionally, we identified a potential interaction between GRP94 and the TGFβ docking receptor LRRC33, a paralog of GARP in M2 macrophages. These results align with our previous findings showing that GRP94 inhibition can reduce tumor volumes and intra-tumoral M2-like macrophages in a murine TNBC model. Taken together, our findings support the hypothesis that GRP94 plays a central role in regulating the TGFβ maturation pathway, not only in regulatory T cells (Tregs) as previously reported, but also in M2 macrophages and tumor cells. These findings further highlight the therapeutic potential of GRP94 inhibition in cancer treatment.

## Materials and methods

### Human PBMC-derived macrophages obtention and cell culture

Peripheral blood mononuclear cells (PBMC) were separated from buffy coats (obtained from Etablissement Français du Sang) using a Ficoll gradient method. Briefly, blood was diluted half in PBS, added on Ficoll (CMSMSL01-01, Eurobio Scientific, Les Ulis, France) (2:1 v:v) and centrifugated 20 min at 700 g with low acceleration and no braking. Then, separated PBMC were washed and remaining red cells were lysed (lysis buffer: 150 mM NH_4_Cl, 10 mM KHCO_3_ and 0,1 mM EDTA). PBMC were seeded and incubated 1 h in culture conditions (i.e. 5% CO_2_, 37 °C). After adhesion, monocytes were differentiated in M2 macrophages with 100 ng/mL macrophage colony stimulating factor (M-CSF, 130-096-492, Miltenyi, Bergisch Gladbach, Germany) in RPMI medium with 10% heat inactivated FBS during 6 days, with differentiation medium renewal after 3 days. Macrophages were then activated with 20 ng/mL interleukine-4 (IL-4, 130-093-922, Miltenyi) in serum free Opti-MEM medium for 24 h. The GRP94 inhibitor PU-WS13 (HY-18680, MedChemExpress, Monmouth Junction, New Jersey, USA) was added 24 h before activation and during the whole activation period.

Human TNBC cells MDA-MB-231 (HTB-26™, ATCC, Manassas, VA, USA) were grown in DMEM with 10% heat inactivated FBS in previously mentioned culture conditions (i.e., 5% CO_2_, 37 °C) and tested for mycoplasma contamination before use. For experiments, cells were seeded 24 h in growth conditions before being treated with GRP94 inhibitors, PU-WS13 or GRP94 inhibitor-1 (HY-112910, MedChemExpress), during 24 h in serum free DMEM.

### Binding affinity assay by microscale thermophoresis (MST)

Specific binding between recombinant human furin (1503-SE, R&D Systems®, USA) and recombinant human GRP94 (RD172379100, Biovendor®, Czech Republic) was measured by MST assays. Briefly, recombinant furin was serially diluted in PBS-Tween 20 0.01% buffer from a maximal furin concentration at 3.58 μM to 0.1 nM and Nano-RED-labelled recombinant GRP94 was added at a constant concentration of 100 nM for each furin dilution point. A negative control was also performed with the same procedure by replacing furin with a recombinant human Hsp110 kindly provided by Dr. François Hermetet (Inserm UMR 1231, Dijon, France) diluted from 2.33 μM to 0.07 nM. The mixtures were loaded into Monolith NT.115 Capillaries (NanoTemper Technologies, Germany) and thermophoresis was performed using a Monolith NT.115 (NanoTemper Technologies, Germany) at room temperature with excitation power and MST power both at 40%. The data were analyzed using MO. Affinity Analysis v2.3 software (NanoTemper Technologies, Germany).

### Furin enzymatic activity assay

After treatment, PBMC-derived M2 macrophages or MDA-MB-231 cells were harvested by scrapping, washed with PBS and lysed using a TRITON X-100 based lysis buffer (20 mM Tris-HCl, 150 mM NaCl, 1% Triton X-100, H_2_O). Cell lysate (20 μL) was diluted in 20 μL of ultrapure H_2_O and 1 nmol of a fluorogenic substrate to furin: Pyr-Arg-Thr-Lys-Arg-AMC trifluoroacetate salt (4018149, Bachem, Bubendorf, Switzerland). Furin enzymatic activity was determined by recording fluorescence (355 nm excitation and 460 nm emission) over time using an EnVision® multimode plate reader (PerkinElmer®, Waltham, Massachusetts, USA).

### Western blotting

PBMC-derived M2 macrophages or MDA-MB-231 cells were harvested by scrapping, washed with PBS and lysed 20 min at 4 °C in cell lysis buffer (#9803, Cell Signaling Technology, Danvers, Massachusetts, USA) supplemented with Complete™ Protease Inhibitor Cocktail (11697498001, Merck, Darmstadt, Germany) and phosphatase inhibitor cocktails 2 and 3 (P5726 and P0044, Sigma-Aldrich, Saint-Louis, Missouri, USA). After centrifugation 15 min at 14,000 g to remove cellular debris, samples were prepared in classical loading buffer, heated 5 min at 95 °C, loaded on SDS-PAGE gels before being transferred on PVDF membranes and saturated with 3% BSA diluted in TBS-0,1% Tween-20. PVDF membranes were incubated with primary antibodies (diluted at 1:1000 in saturation buffer) overnight under constant agitation at 4 °C and, after washing, with the corresponding HRP-conjugated secondary antibody (712-036-153, Jackson Immunol Research, Cambridge, UK; #7074, Cell Signaling Technology, Danvers, Massachusetts, USA; 1:10000 dilution in saturation buffer) 1 h at room temperature. Primary antibodies: GRP94 (ADI-SPA-850, Enzo Life Sciences, Villeurbanne, France), TGF-β1 (ab215715, Abcam, Cambridge, UK), Furin (ab183495, Abcam), MMP14 (AB6004, Merck). Mouse monoclonal Anti-β-Actin−Peroxidase antibody (A3854, Sigma-Aldrich) was used for loading control.

For western blotting on supernatants, cell culture supernatants were collected, centrifugated 5 min at 400 g to remove remaining cells and concentrated using Amicon® Ultra-0.5 Centrifugal Filter Devices with a 3 kDa molecular weight cut-off (UFC500396, Merck) following the manufacturer’s instructions. Briefly, 500 µL supernatants were added on filter devices, centrifugated 30 min at 14,000 g and finally turned upside down in clean tubes and centrifugated again 2 min at 1000 g to collect concentrated supernatants. Samples were then prepared for SDS-PAGE western blotting as described above.

### GRP94-Furin co-immunoprecipitation assay

PBMC-derived M2 macrophages or MDA-MB-231 cells were harvested by scrapping, washed with PBS and lysed 20 min at 4 °C in cell lysis buffer (#9803, Cell Signaling Technology, Danvers, Massachusetts, USA) supplemented with Complete™ Protease Inhibitor Cocktail (11697498001, Merck, Darmstadt, Germany). After centrifugation 15 min at 14,000 g to remove cellular debris, samples were incubated overnight at 4 °C with 4 µg of anti-GRP94 (ab3674, Abcam) or a non-relevant antibody (X0903, Dako, Santa Clara, California, USA). Then, samples were incubated 30 min at 4 °C with 100 µL of µMACS™ Protein A MicroBeads (130-071-001, Miltenyi) and loaded on µColumns (130-042-701, Miltenyi) placed on a magnetic µMACS™ Separator (130-042-602, Miltenyi). Samples were washed 4 times with lysis buffer, incubated 5 min with 20 µL loading buffer heated at 95 °C and eluted using 50 µL of hot loading buffer. Finally, samples were heated 5 min at 95 °C and SDS-PAGE western blotting was performed as described above. Anti-vinculin antibody (V9131, Merck) was used for input loading control.

### Immunofluorescence

PBMC-derived M2 macrophages seeded on coverslips were washed with PBS, fixed with a 4% formaldehyde solution for 15 min and washed again with PBS. Autofluorescence was then quenched with 50 mM NH_4_Cl for 10 min. After being washed, cells were permeabilized with PBS - 0.1% TRITON X-100, blocked for 1 h with PBS-1% BSA and incubated with primary antibodies diluted at 1:100 in PBS-1% BSA at 4 °C overnight: anti-GRP94 (ab210960, Abcam) and anti-LRRC33 (PA5-23822, Invitrogen, Waltham, Massachusetts, USA). Then cells were washed and stained 30 min at room temperature with secondary fluorescent antibodies (A11008 and A11004, Invitrogen) diluted at 1:1000 in PBS-1% BSA. After washes, coverslips were mounted in ProLong® Gold Antifade Reagent with DAPI (P36941, ThermoFisher Scientific, Waltham, Massachusetts, USA). Microscopy images were acquired using an Axio Imager 2 equipped with an AxioCam MRm monochrome CCD camera (Carl Zeiss Microscopy GmbH, Jena, Germany) and analyzed with Zen 2.3 Lite® software (Carl Zeiss Microscopy GmbH, Germany).

### Duolink® Proximity-Ligation Assay (PLA™)

PLA™ was performed with Duolink® In Situ Detection Reagents Orange kit (DUO92007, Sigma-Aldrich). Cells seeded on coverslips were washed twice with PBS and fixed in 4% formaldehyde solution for 10 min at 4 °C. They were then permeabilized and saturated with a PBS - 3% BSA - 0.2% saponin solution for 20 min at room temperature before being incubated overnight at 4 °C with primary antibodies couples diluted at 1:100 in saturation buffer: anti-GRP94 (ab210960 or ab3674, Abcam) and one of the following antibodies: anti-Furin (ab183495, Abcam), anti-LRRC33 (PA5-23822, Invitrogen) or anti-HSP105 (sc-74550, SantaCruz Biotechnology, Dallas, Texas, USA). After incubation, cells were washed with PBS and incubated with Duolink® In Situ PLA® Probe Anti-Rabbit PLUS (DUO92002, Sigma-Aldrich) and Duolink® In Situ PLA® Probe Anti-Mouse MINUS (DUO92004, Sigma-Aldrich) for 1 h at 37 °C. Thereafter, PLA™ protocol was performed following the manufacturer’s protocol. Finally, slides were mounted in ProLong® Gold Antifade Reagent with DAPI (P36941, ThermoFisher Scientific). Microscopy images were acquired using an Axio Imager 2 equipped with an AxioCam MRm monochrome CCD camera (Carl Zeiss Microscopy GmbH) and analyzed by Zen 2.3 Lite® software (Carl Zeiss Microscopy GmbH). Spots quantification was performed using Icy® 2.0 software (BioImage Analysis Lab, Institut Pasteur, France).

### MDA-MB-231 cell migration analysis using wound healing assay

MDA-MB-231 cells were seeded in a 96-wells plate and cultured during 24 h in growth conditions, then starved with DMEM - 0,5% FBS during 24 h. After starving, the wound was realized using an Incucyte® WoundMaker 96-Tool (4563, Sartorius, Göttingen, Germany). Then, cells were washed with HBSS and treatments with the GRP94 inhibitors, PU-WS13 or GRP94 inhibitor-1, added in serum free DMEM. Finally, cell migration in wound areas was measured during 24 h in culture conditions (5% CO_2_, 37 °C) using an Incucyte® S3 Live-Cell Analysis System (Sartorius) and relative wound densities over time calculated using Incucyte® Scratch Wound Analysis Software Module (9600-0012, Sartorius).

### Cytotoxicity assay

Cytotoxicity of PU-WS13 and GRP94-inhibitor-1 on human primary M2 macrophages and MDA-MB-231 cells was assessed using the MTS Cell Proliferation Assay Kit (ab197010, Abcam) following the manufacturer’s instructions. Cells treated with 10 or 20% DMSO, respectively for MDA-MB-231 or M2 macrophages, were used as positive controls.

### TGF-β1 and MMP14 mRNA transcription analysis by RT-qPCR

After treatment, PBMC-derived M2 macrophages total RNA was extracted using ReliaPrep RNA Miniprep Systems (Z6011, Promega, Madison, Wisconsin, USA). Reverse transcription was performed using the Maxima First Strand cDNA Synthesis Kit for RT-qPCR, with dsDNase (#K1672, ThermoFisher Scientific), and qPCR was performed using the iTaq Universal SYBR Green Supermix (1725121, Bio-Rad, Hercules, California, USA) in a ViiA 7 Real-Time PCR System (ThermoFisher). Experiments were performed following manufacturer’s instructions. Primers for qPCR were purchased from Qiagen (Hilden, Germany): Hs_GAPDH_1_SG QuantiTect Primer Assay (QT00079247, 249900), Hs_TGFB1_1_SG QuantiTect Primer Assay (QT00000728, 249900), and Hs_MMP14_1_SG QuantiTect Primer Assay (QT00001533, 249900).

### Statistical analysis

Statistical analysis of data was performed using GraphPad Prism 10.2.3 software (La Jolla, CA, USA). Shapiro-Wilk test was used to assess data compliance with normal homogeneity of variance. Western-blot quantification and cytotoxicity data were analyzed through One-way ANOVA with Friedman test using the two-stage linear step-up procedure of Benjamini, Krieger and Yekutieli. PLA spots quantification data were analyzed using two-tailed Mann-Whitney test. RT-qPCR fold increase data were analyzed using two-tailed Wilcoxon test. Two-way Anova was used for furin enzymatic activity and relative wound densities with Tukey’s multiple comparison test. Western-blot quantifications were normalized by the non-treated condition and are presented as a fold change. Data are presented as means + /− SEM (standard error of the mean). *P*-values below 0.05 were considered statistically significant.

## Supplementary information

Supplementary Figure legends

Figure S1: Validation of M2 macrophage differentiation

Figure S2: PU-WS13 cytotoxicity on M2 macrophages

Figure S3: GRP94 inhibitor PU-WS13 does not impact TGFβ nor MMP14 mRNA transcription in M2 macrophages

Figure S4: GRP94 inhibitor PU-WS13 does not impact furin expression in M2 macrophages

Figure S5: GRP94 inhibitors cytotoxicity on MDA-MB-231 cells

Original blots

## Data Availability

All data supporting the findings of this study are available upon request to the corresponding author.
