# Supplementary material for: The chaperone GRP94 interacts with the proprotein convertase furin and regulates TGF-beta maturation in human primary M2 macrophages
Source: Cell Death Discov. 2025 Dec 15;11:558. doi: 10.1038/s41420-025-02866-2 (PMC12706012; doi:10.1038/s41420-025-02866-2)
Supplement: Supplementary file 1 — Supplementary Figure legends [file 41420_2025_2866_MOESM1_ESM.docx]

**Supplementary figures legends**

**Figure S1: *Validation of M2 macrophage differentiation***

Western-blot analysis of STAT6 phosphorylation in human PBMC-derived M0 (M-CSF) and M2 macrophages (M-CSF + IL-4) (representative images, n=8) (* p < 0.05).

**Figure S2: *PU-WS13 cytotoxicity on M2 macrophages***

Cell viability was measured through MTS assay in M2 macrophages treated or not with PU-WS13 12.5 or 25 µM during 24h. Treatment by DMSO 20 % was used as positive control. (n=8) (*** p < 0.001).

**Figure S3: *GRP94 inhibitor PU-WS13 does not impact TGFβ nor MMP14 mRNA transcription in M2 macrophages***

RT-qPCR analysis of TGFβ1 (left panel) and MMP14 (right panel) mRNA transcription in human PBMC-derived M2 macrophages treated or not with PU-WS13 25 µM. Data are represented as fold increase versus non treated condition (n=4).

**Figure S4: *GRP94 inhibitor PU-WS13 does not impact furin expression in M2 macrophages***

Western-blot analysis of furin expression in human PBMC derived M2 macrophages treated or not with PU-WS13 12.5 or 25 µM during 24h. (representative images, n=10) (* p < 0.05).

**Figure S5: *GRP94 inhibitors cytotoxicity on MDA-MB-231 cells***

Cell viability was measured through MTS assay in MDA-MB-231 cells treated or not with PU-WS13 12.5 or 25 µM and GRP94 inhbitor-1 2.5, 5 or 10 µM during 24h. Treatment by DMSO 10 % was used as positive control. (n=5) (* p < 0.05).
