# Supplementary figures and images for: The chaperone GRP94 interacts with the proprotein convertase furin and regulates TGF-beta maturation in human primary M2 macrophages
Source: Cell Death Discov. 2025 Dec 15;11:558. doi: 10.1038/s41420-025-02866-2 (PMC12706012; doi:10.1038/s41420-025-02866-2)

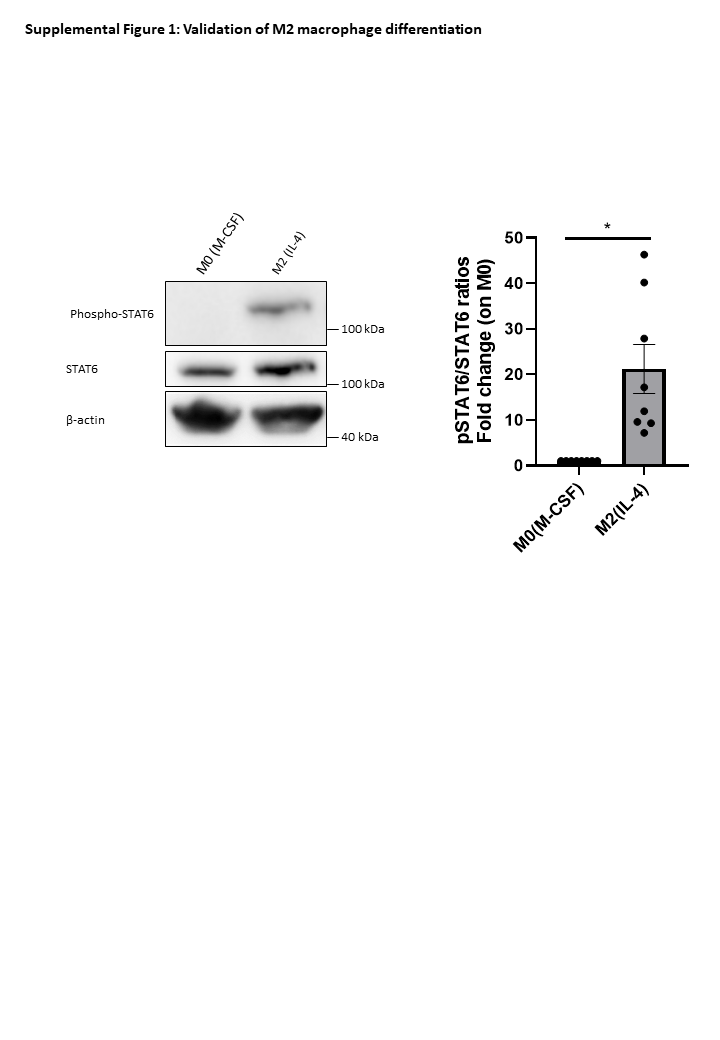

Supplement: Supplementary file 2 — Figure S1: Validation of M2 macrophage differentiation [file 41420_2025_2866_MOESM2_ESM.png]

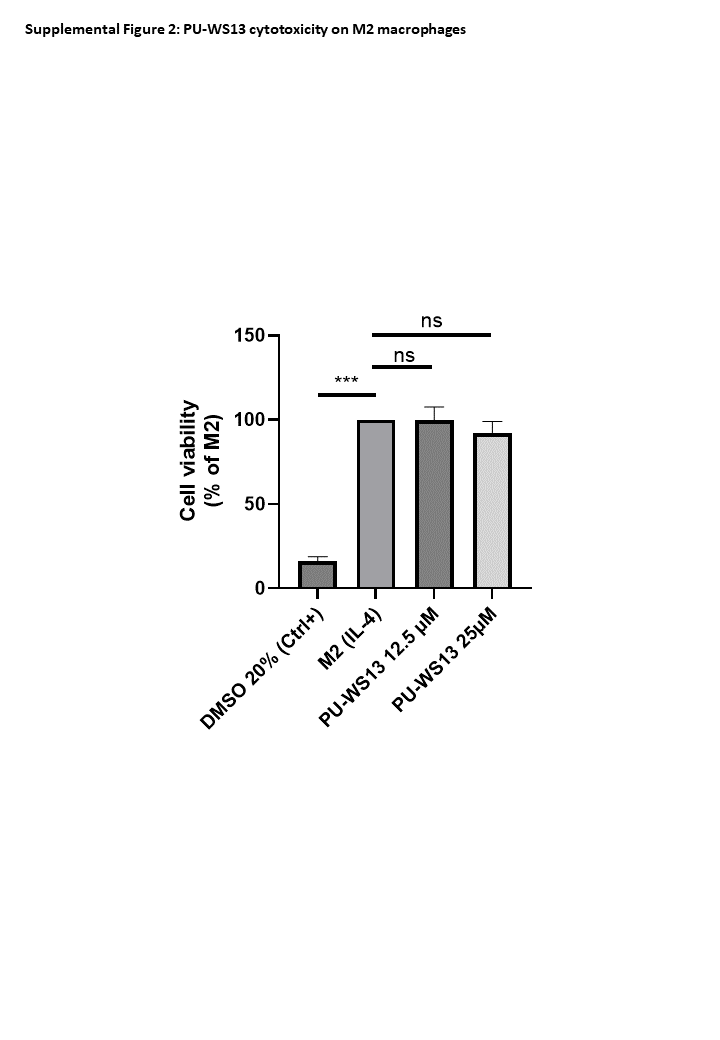

Supplement: Supplementary file 3 — Figure S2: PU-WS13 cytotoxicity on M2 macrophages [file 41420_2025_2866_MOESM3_ESM.png]

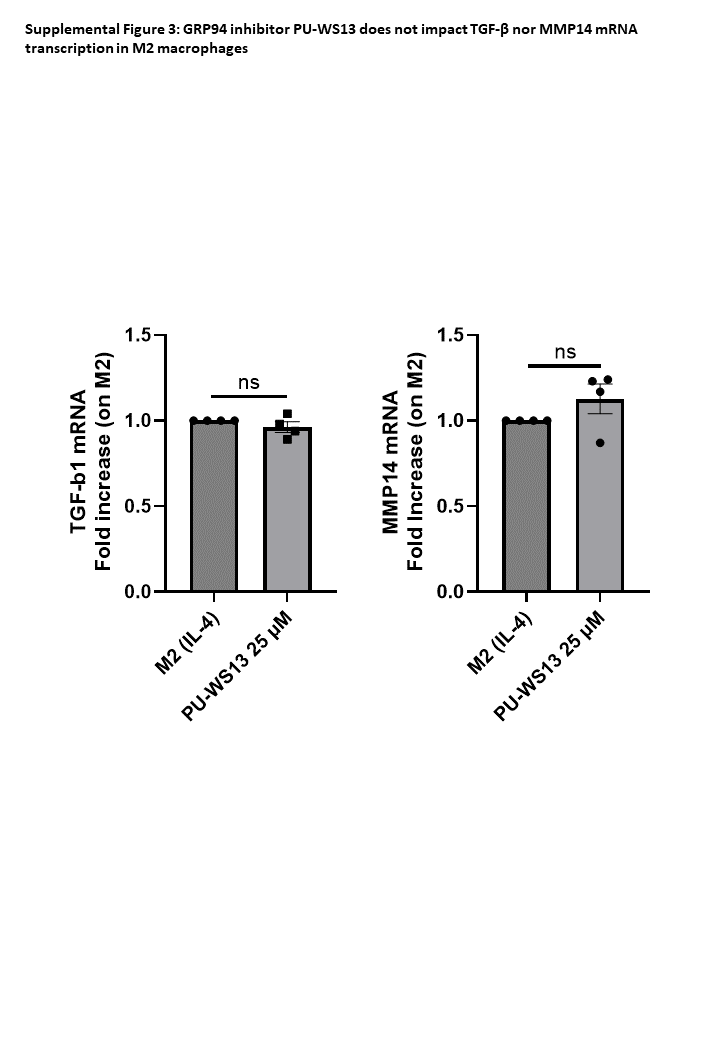

Supplement: Supplementary file 4 — Figure S3: GRP94 inhibitor PU-WS13 does not impact TGFβ nor MMP14 mRNA transcription in M2 macrophages [file 41420_2025_2866_MOESM4_ESM.png]

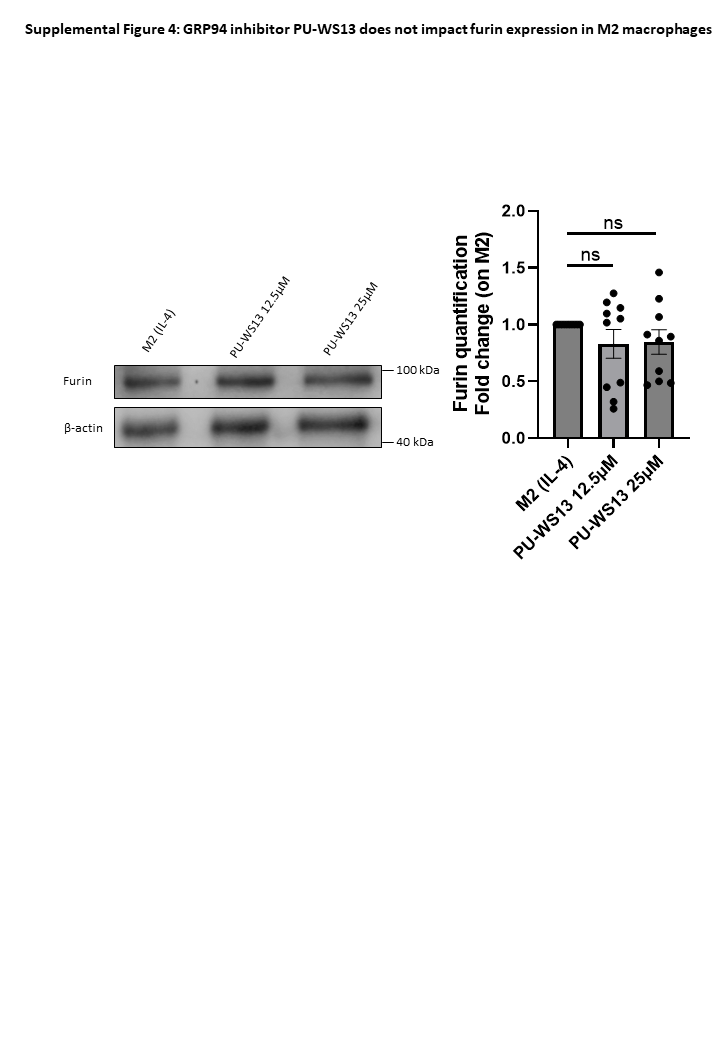

Supplement: Supplementary file 5 — Figure S4: GRP94 inhibitor PU-WS13 does not impact furin expression in M2 macrophages [file 41420_2025_2866_MOESM5_ESM.png]

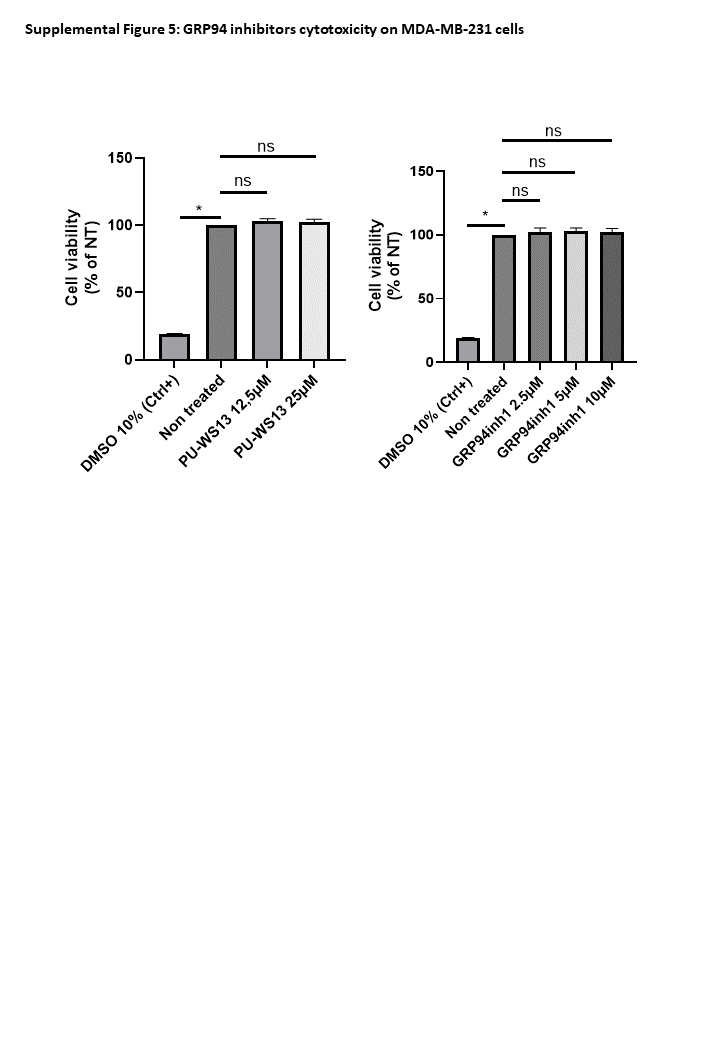

Supplement: Supplementary file 6 — Figure S5: GRP94 inhibitors cytotoxicity on MDA-MB-231 cells [file 41420_2025_2866_MOESM6_ESM.png]

Figure 1A

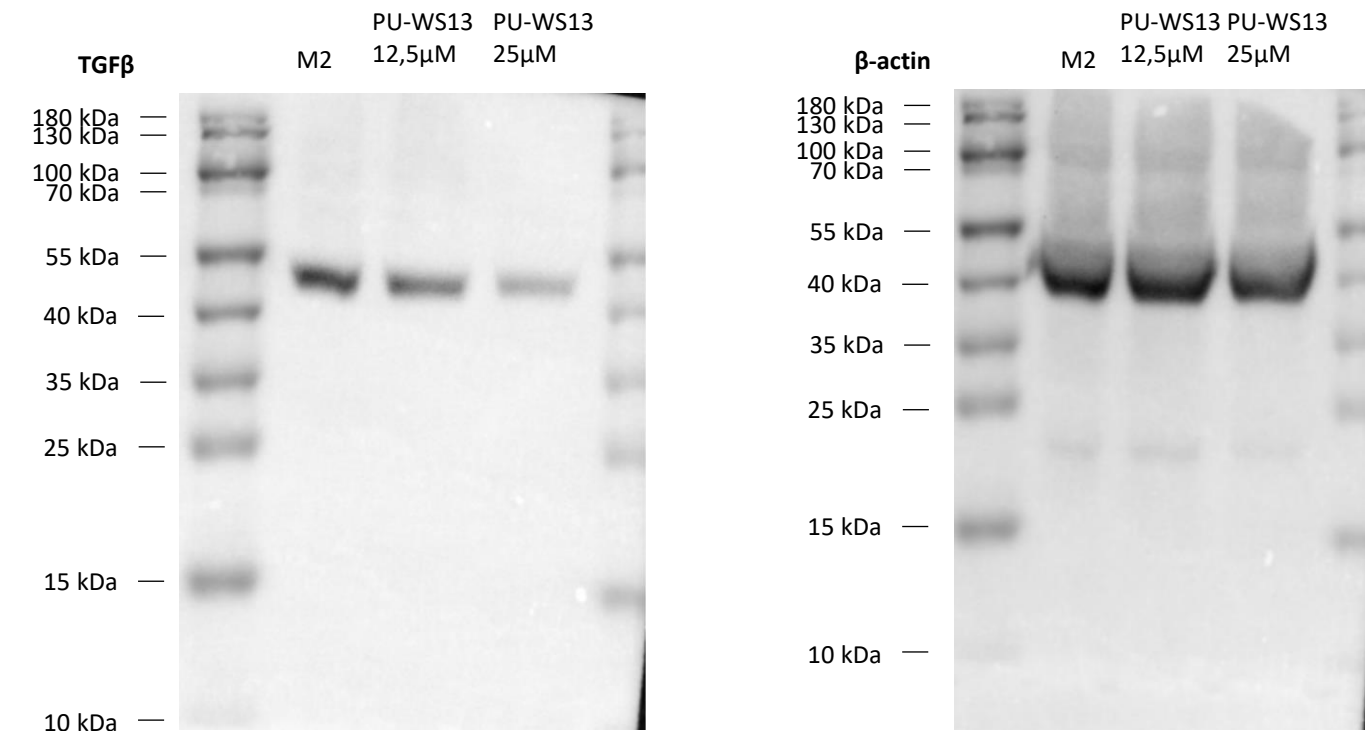

Figure 1B

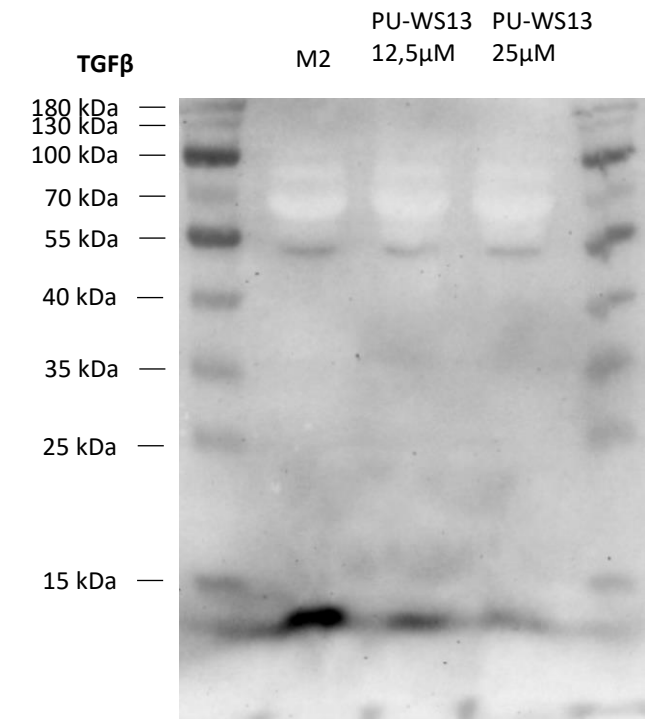

Figure 1D

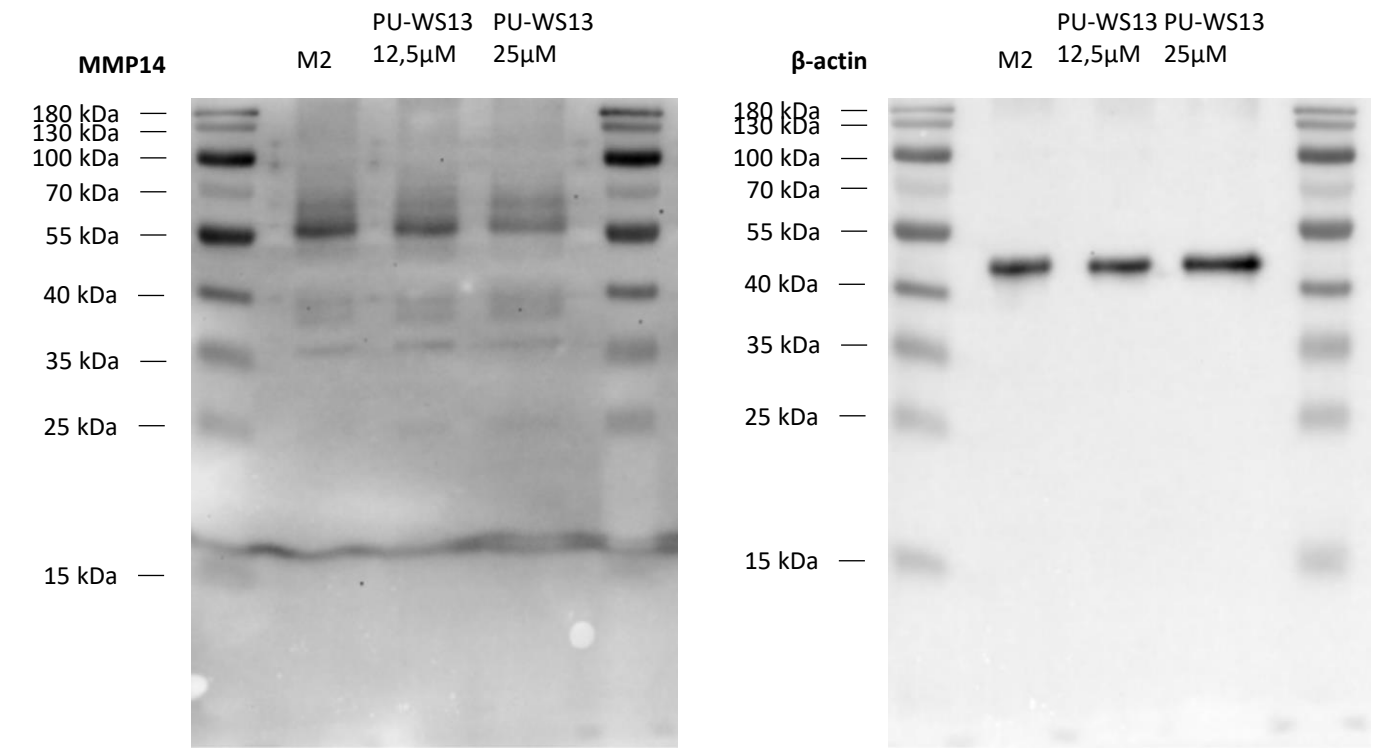

Figure 2C

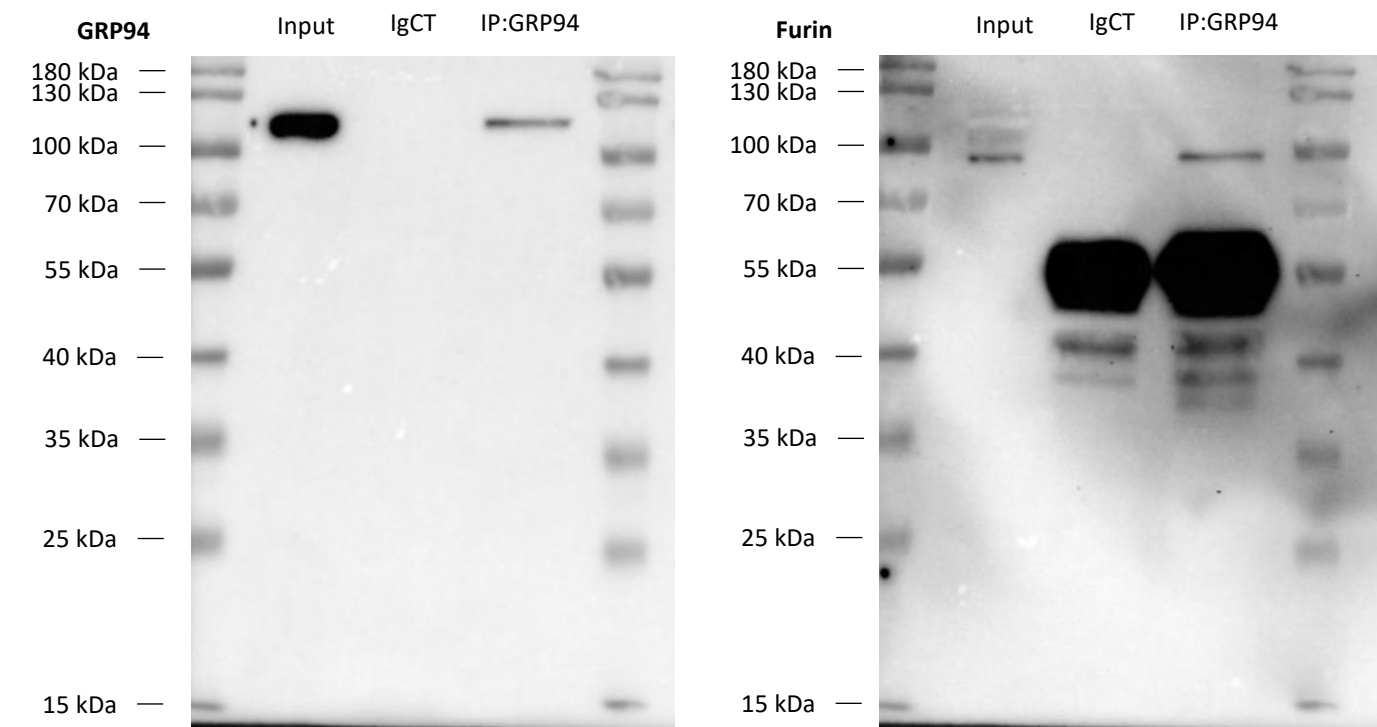

Figure 2C

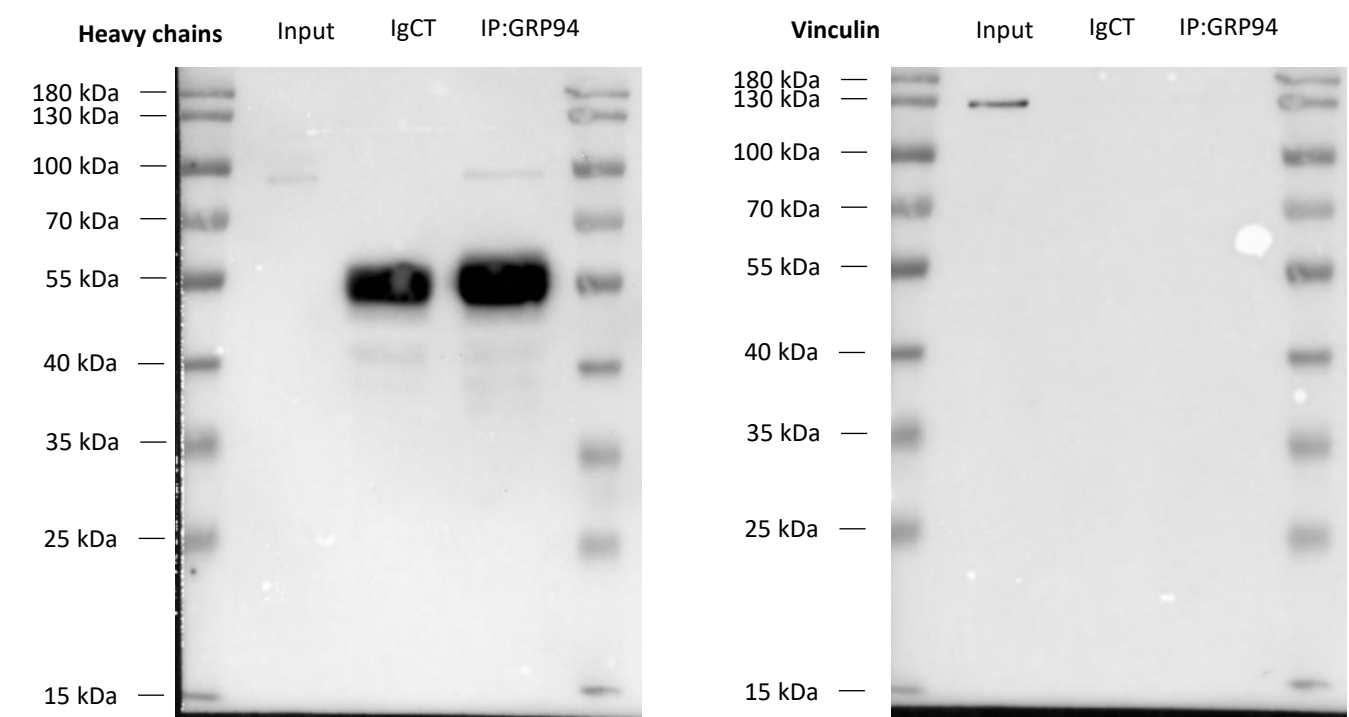

Figure 3B

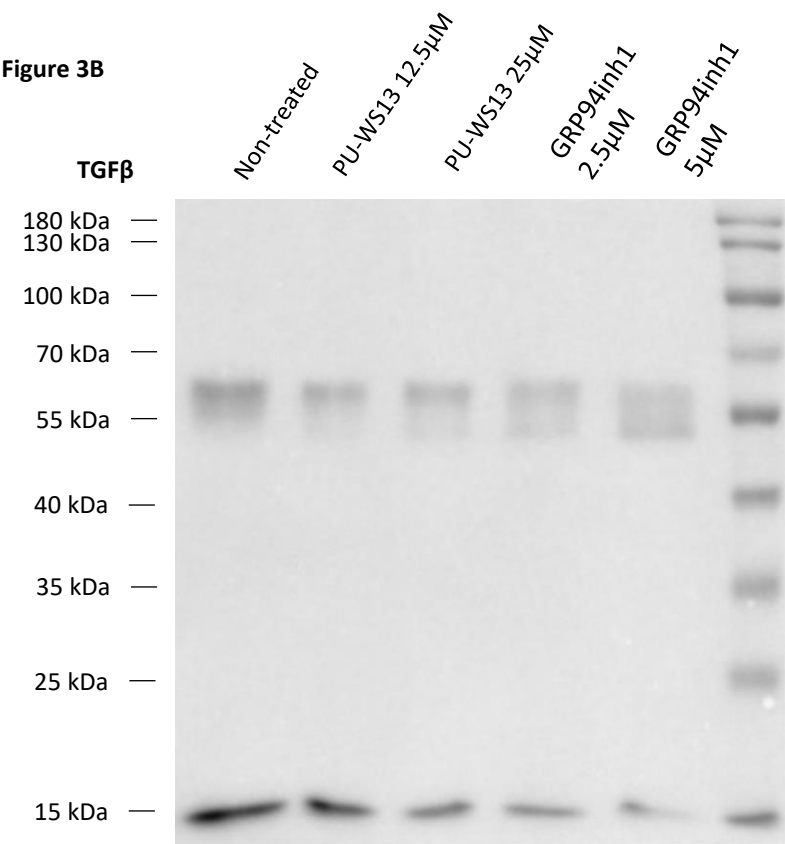

Figure 3C

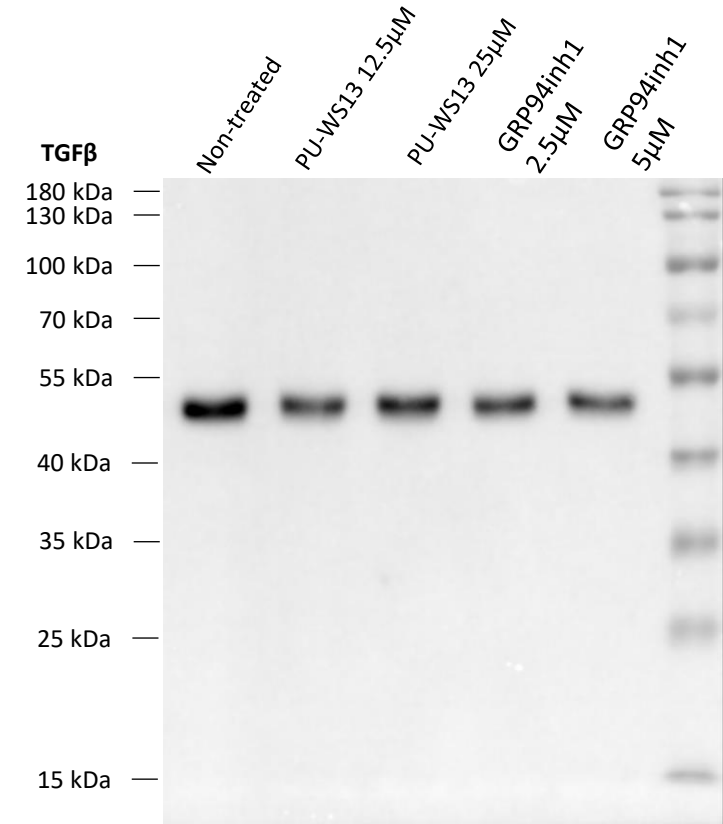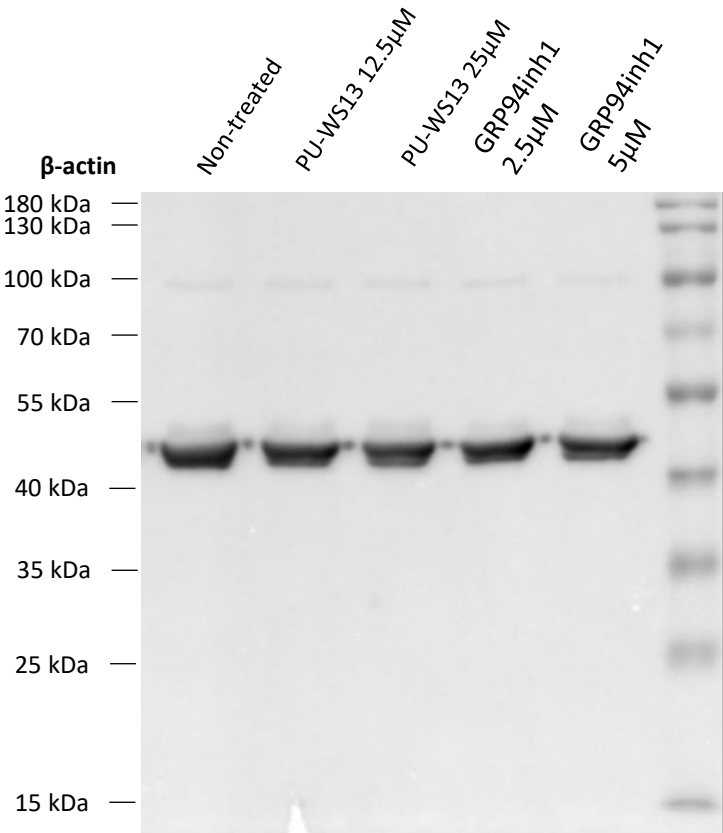

Figure 3D

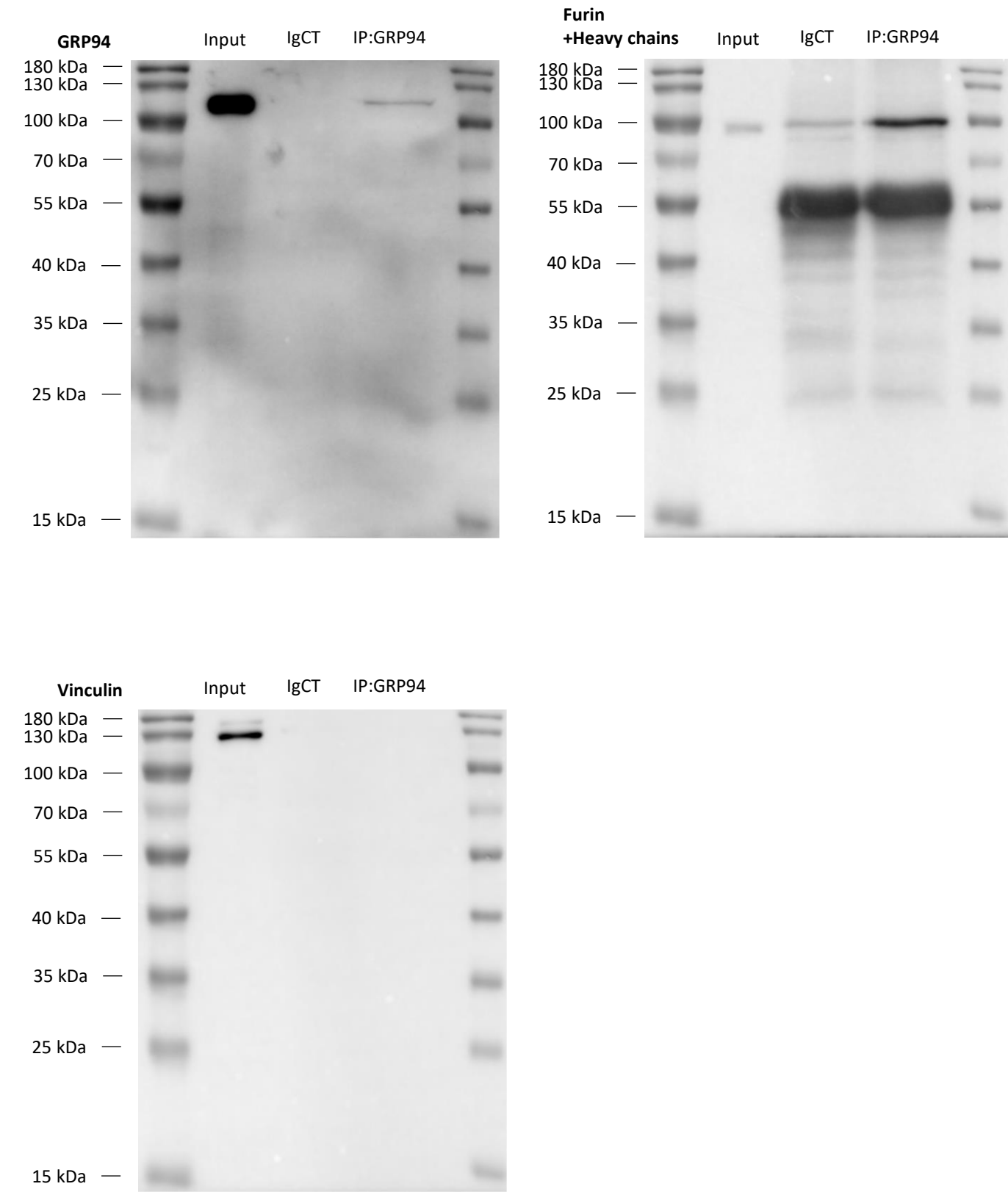

Figure S1

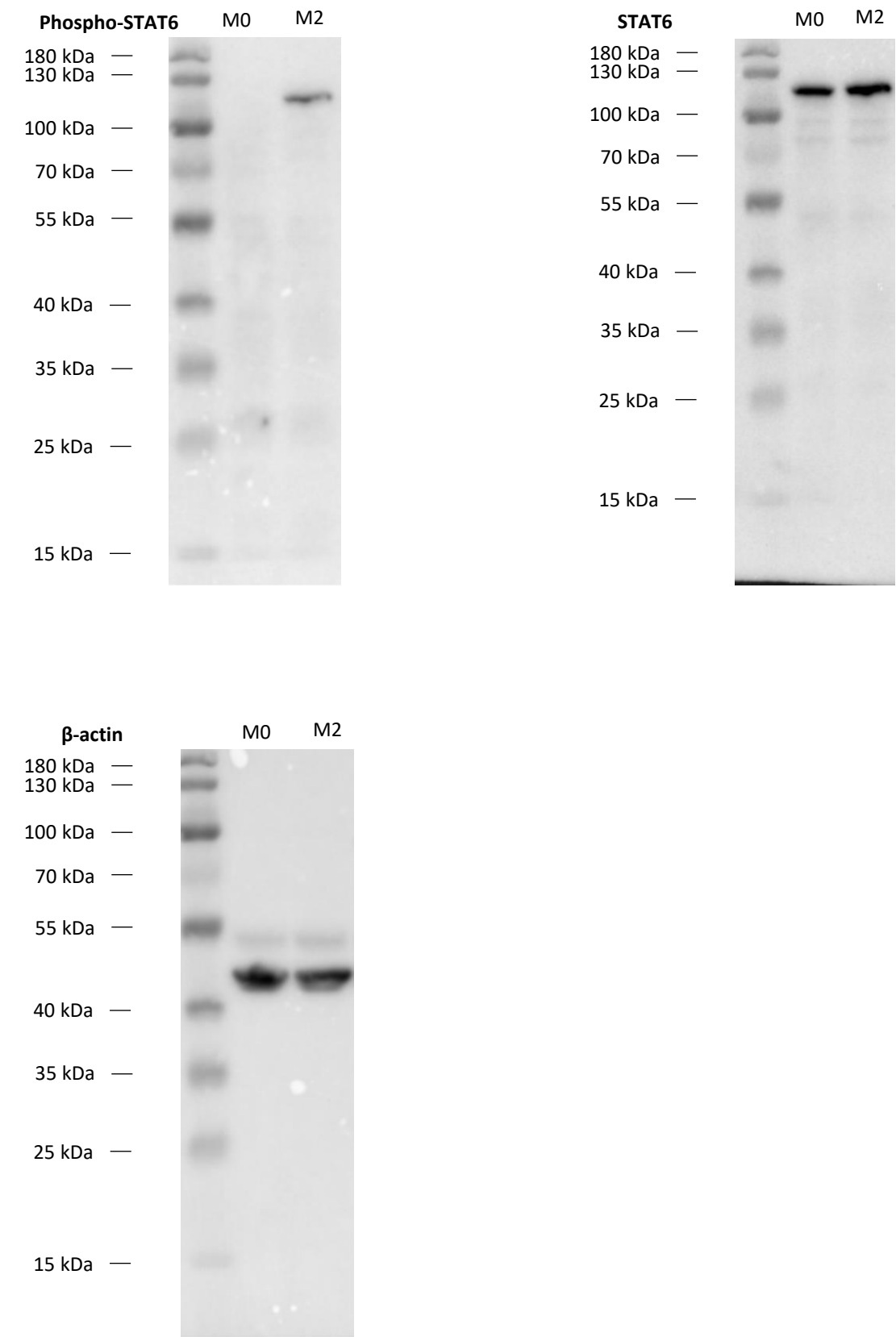

Figure S4

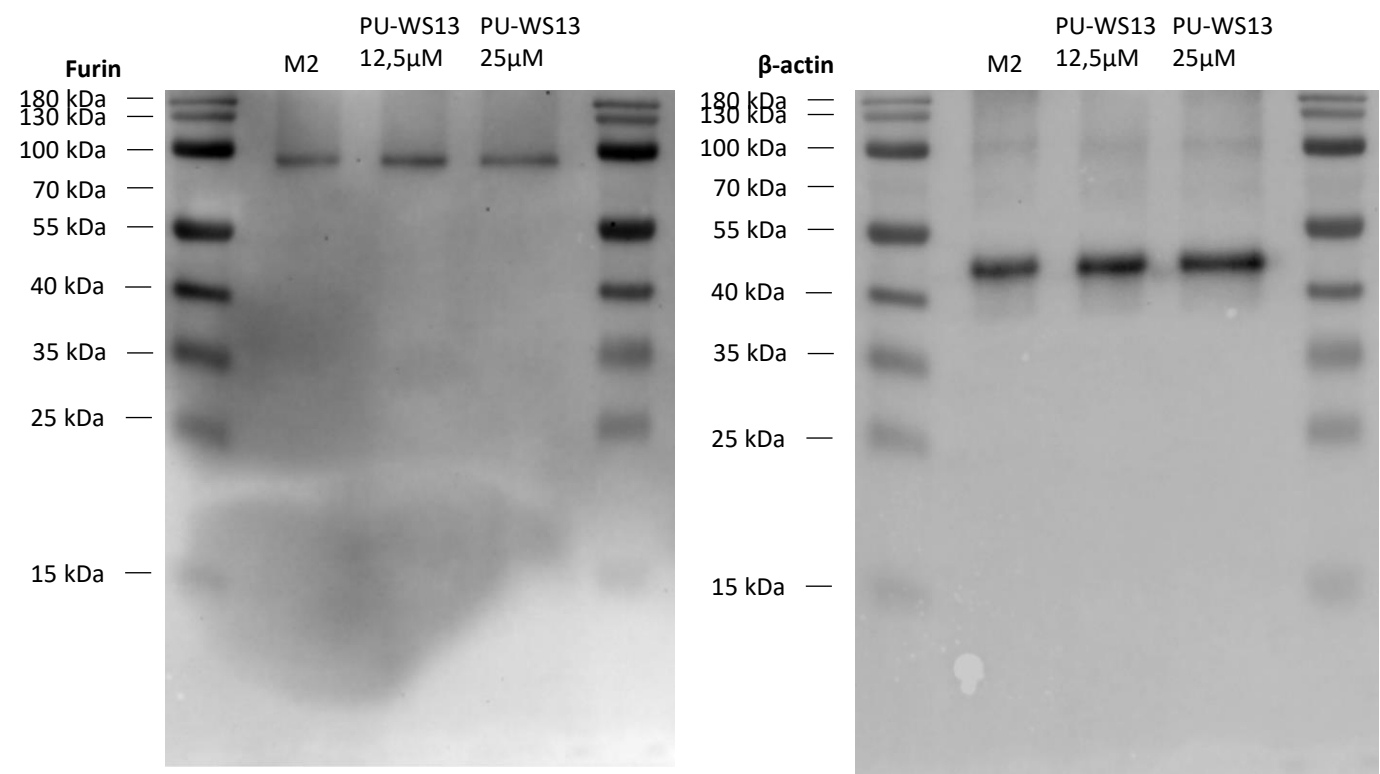

Supplement: Supplementary file 7 — Original blots [file 41420_2025_2866_MOESM7_ESM.pdf]
